# Electron-injected Pd@CeO_2_ nanozymes for multifaceted ROS scavenging and protection against ischemia-reperfusion injury in skin flaps

**DOI:** 10.1186/s12951-025-03775-3

**Published:** 2025-10-21

**Authors:** Lingling Zhou, Jiayuan Sun, Tianxiang Lu, Xinya Zhang, Mingkang Wang, Yanjun Xu, Jia Zhou, Xiaoyang Li, Wenxian Du, Fan Yang, Yuehua Li

**Affiliations:** 1 Institute of Diagnostic and Interventional Radiology, Shanghai Sixth People’s Hospital Affiliated to Shanghai Jiao Tong University School of Medicine, Shanghai, 200233 People’s Republic of China; 2 Faculty of Medical Imaging Technology, College of Health Science and Technology, School of Medicine, Shanghai Jiao Tong University, Shanghai, People’s Republic of China; 3 Department of Pharmacy, Renji Hospital, School of Medicine, Shanghai Jiao Tong University, Shanghai, 200127 People’s Republic of China; 4 Department of Obstetrics and Gynecology, Xijing Hospital, Affiliated to the Fourth Military Medical University, Xian, 710032 People’s Republic of China; 5 Department of Plastic and Reconstructive Surgery, School of Medicine, Ninth People’s Hospital Affiliated to Shanghai Jiao Tong University, Shanghai, 200011 People’s Republic of China; 6 Wuhan United Imaging Life Science Instruments Ltd, Wuhan, 430000 People’s Republic of China; 7 Jiangsu Health Vocational College, Nanjing, 210000 People’s Republic of China; 8 Department of Ultrasound in Medicine, School of Medicine, Shanghai Sixth People’s Hospital Affiliated to Shanghai Jiao Tong University, Shanghai, 200233 People’s Republic of China; 9 School of Agriculture and Biology, Shanghai Jiao Tong University, 800 Dongchuan Road, Shanghai, 200240 People’s Republic of China

**Keywords:** Nanoregenerative medicine, CeO_2_ nanozyme, ROS, Organ injury repair, Electron injection, Ischemia-reperfusion injury

## Abstract

Ischemia–reperfusion (I/R) injury in skin flap transplantation causes acute oxidative damage and inflammation, leading to high failure rates and tissue necrosis. Herein, we present a core–shell Pd@CeO_2_ nanozymes that addresses this challenge via a unique electron-injection mechanism across the Pd–CeO_2_ interface, conferring unprecedented antioxidant and anti-inflammatory potency. Constructed by confining Pd clusters within a ceria matrix, this nanozyme combines catalase- and superoxide dismutase–like catalytic activities for robust reactive oxygen species (ROS) scavenging. In vitro, Pd@CeO_2_ rapidly neutralizes ROS, preventing oxidative cell death by reducing apoptosis and dampening inflammatory signaling, while restoring angiogenic potential. In a rat skin flap I/R model, Pd@CeO_2_ significantly improves flap survival and microvascular regeneration while concurrently reducing tissue necrosis, apoptosis, and inflammation, with no observable toxicity. By simultaneously alleviating oxidative stress, cell death, inflammation, and vascular dysfunction, this nanozyme offers a comprehensive therapeutic strategy against I/R injury. This work introduces a new paradigm for nanozyme-based cytoprotection in transplantation, with potential applicability to other ischemic injuries.

**Supplementary Information:**

The online version contains supplementary material available at 10.1186/s12951-025-03775-3.

## Introduction

Skin flap transplantation is a cornerstone of reconstructive surgery for repairing extensive tissue defects arising from trauma, oncologic resection, and congenital anomalies [1, 2]. Despite surgical advances, ischemia-reperfusion (I/R) injury remains a significant clinical hurdle, frequently causing partial or total flap necrosis and severe postoperative complications [3–5]. I/R injury is characterized by excessive reactive oxygen species (ROS) generation during reperfusion, leading to oxidative stress, inflammatory cascades, endothelial dysfunction, apoptosis, and microvascular impairment [4, 6–8]. Collectively, these pathological processes severely compromise tissue viability [9], adversely affecting clinical outcomes and patient recovery.

Current preventive strategies, including ischemic preconditioning, hyperbaric oxygen therapy, and systemic antioxidants, provide limited and often inconsistent protection due to strict timing requirements, poor bioavailability, rapid degradation, and inadequate targeting of ROS within ischemic tissues [10–15]. These limitations underscore a critical unmet clinical need for innovative, robust therapeutic strategies capable of simultaneously addressing the multifaceted pathology of I/R injury.

Nanozyme-based therapies have recently emerged as promising alternatives due to their enzyme-like catalytic activities, high stability, and tunable properties [4, 16, 17]. Compared to traditional antioxidants and natural antioxidant enzymes, nanozymes offer advantages such as low cost, excellent stability, and ease of synthesis, overcoming the limitations of natural enzymes, including poor bioavailability and rapid degradation [18–20]. By mimicking the catalytic activities of natural antioxidant enzymes such as superoxide dismutase (SOD) and catalase (CAT), nanozymes efficiently neutralize excessive ROS and restore redox balance [21]. Cerium oxide (CeO_2_) nanoparticles, recognized for their SOD-mimetic activity, exhibit ROS-scavenging potential [22–24], but their performance is often constrained under severe oxidative stress due to limited catalytic efficiency and inadequate substrate specificity [25].

**Scheme 1:**
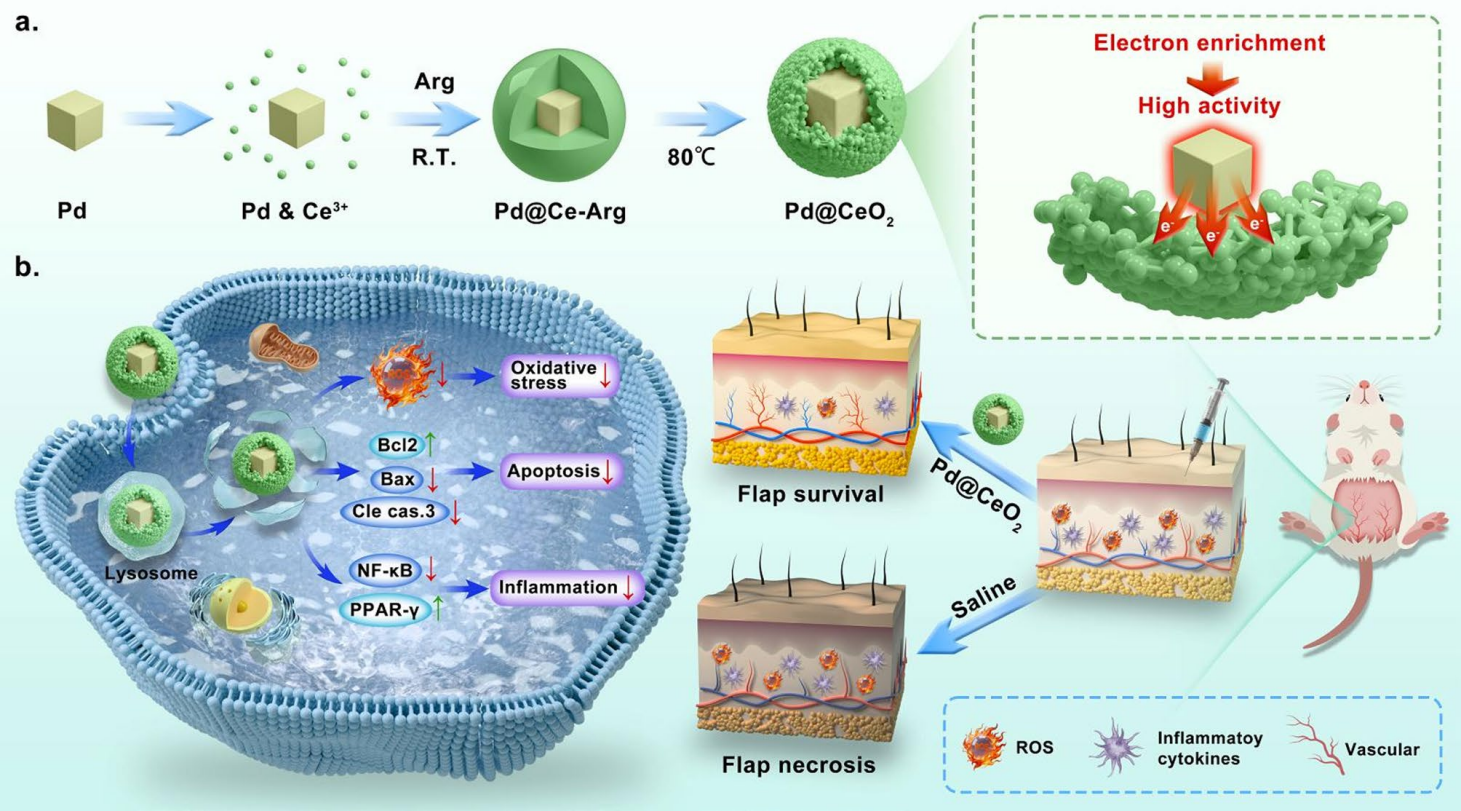
Mechanistic schematic illustrating the role of Pd@CeO_2_ in mitigating oxidative stress, inhibiting apoptosis, and suppressing neuroinflammation

To overcome these limitations, we designed a novel hybrid core-shell nanozyme, Pd@CeO_2_, which leverages electron injection at the Pd–CeO_2_ interface to significantly enhance catalytic activity. The Pd nanoparticles confined within the CeO_2_ shell facilitate strong metal–support interactions, transferring electrons to sustain CeO_2_ in a reduced, highly active catalytic state. This electron injection mechanism markedly enhances both catalase (CAT)-like and SOD-like activities, providing synergistic and efficient ROS neutralization [26–30]. The designed Pd@CeO_2_ can effectively scavenge ROS, protect cells from oxidative damage, suppress apoptosis via the Bax/Bcl-2/caspase-3 signaling pathway, and modulate inflammation through the PPAR-γ/NF-κB pathway. Furthermore, Pd@CeO_2_ enhances vascular regeneration by promoting VEGFA expression and angiogenesis. In a rat skin flap I/R model, Pd@CeO_2_ significantly improves flap survival, reduces inflammation and apoptosis, and promotes microvascular reconstruction (Scheme 1). By combining a biocompatible metal oxide with a catalytically active noble metal, our Pd@CeO_2_ nanozyme not only addresses critical limitations of existing treatments but also establishes a new therapeutic paradigm for managing I/R injury. This approach holds considerable promise for broader applications in tissue transplantation and other oxidative stress-related disorders, potentially transforming clinical practices in reconstructive surgery and regenerative medicine.

## Results and discussion

### Morphological and structural analysis of Pd@CeO_2_

**Fig. 1:**
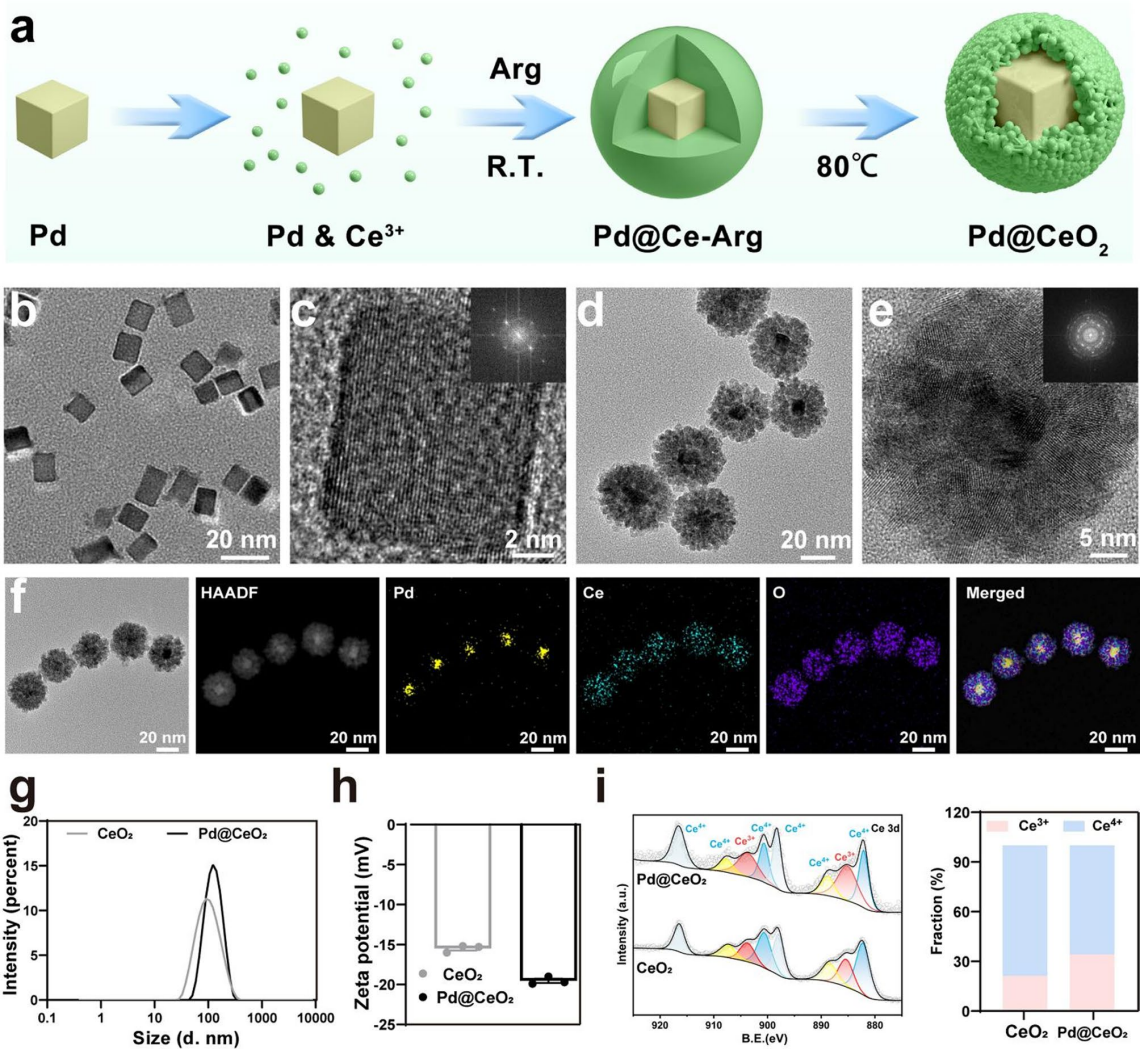
Synthesis and characterization of Pd@CeO_2_ nanozymes. (**a**) Schematic illustration of the Pd@CeO_2_ nanozymes preparation process. (**b**) TEM images of Pd nanocubes. (**c**) Electron diffraction (SAED) pattern of Pd nanocubes. (**d**) TEM images of Pd@CeO_2_ nanozymes. (**e**) SAED pattern of Pd@CeO_2_ nanozymes. (**f**) Element mapping of Pd, Ce, O, and the merged image of these three elements. (**g**-**h**) Particle size distribution and zeta potential of Pd@CeO_2_ nanozymes. (i) XPS of Pd@CeO_2_ nanozymes

Pd@CeO_2_ nanozymes were synthesized via a multi-step process depicted in Fig. 1a. Uniform Pd nanocubes (~ 18 nm) were initially formed by reducing Na_2_PdCl_4_ using ascorbic acid, polyvinylpyrrolidone, and potassium bromide, ensuring monodispersed and precisely sized nanoparticles (Fig. 1b–c). Subsequently, cerium nitrate was introduced in the presence of L-Arginine as a capping agent, facilitating CeO_2_ nanoparticles’ self-assembly onto the Pd nanocube surfaces. This process resulted in a distinct core-shell Pd@CeO_2_ structure, confirmed by TEM imaging showing complete and uniform encapsulation of Pd cores by CeO_2_ shells (Fig. 1d–e). Energy-dispersive X-ray spectroscopy (EDS) elemental mapping (Fig. 1f) demonstrated the spatial distribution of Pd, Ce, and O, further confirming successful core-shell formation. The crystallinity of Pd and Pd@CeO_2_ phases was validated through X-ray diffraction (XRD), which exhibited characteristic peaks corresponding to both materials (Figure S1). Dynamic light scattering (DLS) measurements confirmed that Pd@CeO_2_ nanozymes exhibited a mean particle diameter of 103.9 ± 0.56 nm (Fig. 1g), whereas the zeta potential measurement indicated a surface charge of −19.5 ± 0.47 mV (Fig. 1h).

To elucidate the electron-injection mechanism, X-ray photoelectron spectroscopy (XPS) was employed. Analysis of the Ce 3 d spectra revealed a marked increase in the Ce^3+^/Ce^4+^ ratio in Pd@CeO_2_ (34.23%/65.77%) compared with pristine CeO_2_ (21.29%/78.71%). Furthermore, peak fitting of the Ce 3d spectra revealed that Pd@CeO_2_ exhibits overall lower binding energies than CeO_2_ (Fig. 1i). In particular, the enhanced intensity of Ce^3+^ peaks at these shifted positions indicates electron enrichment at the CeO_2_ interface, arising from interfacial electron transfer from Pd to CeO_2_, which provides robust ROS-scavenging performance. Stability assessments over 7 days revealed minimal particle aggregation or charge alterations, underscoring the nanoparticle’s suitability for physiological applications (Figure S2).

### Enzyme-mimicking activities of Pd@CeO_2_ and mechanistic insights from DFT calculations

**Fig. 2:**
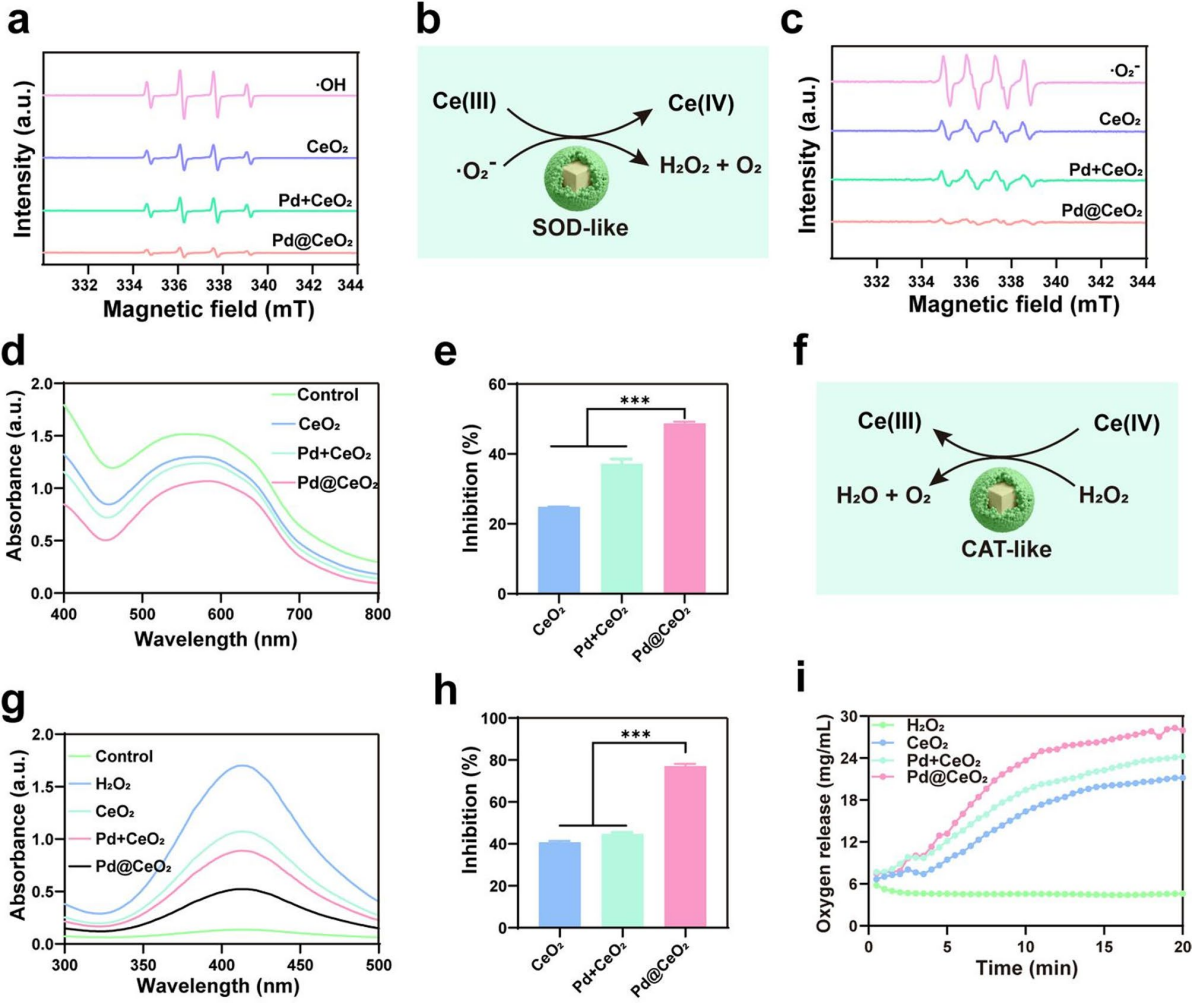
ROS elimination by Pd@CeO_2_ nanozymes. (**a**) ESR spectra of ·OH radicals trapped by DMPO in different samples (positive group, CeO_2_, Pd + CeO_2_ and Pd@CeO_2_). (**b**) Schematic representation of the SOD-like catalytic mechanism of Pd@CeO_2_. (**c**) ESR spectra of ·O_2_^−^ scavenging activity for CeO_2_, Pd + CeO_2_, and Pd@CeO_2_. (**d**) NBT assay showing the reduction of ·O_2_^−^ by Pd@CeO_2_ through decreased absorbance at 560 nm. (**e**) Inhibition rates of ·O_2_^−^ by different formulations. (**f**) Schematic of the CAT-like catalytic mechanism of Pd@CeO_2_. (**g**) CAT-like activity of Pd@CeO_2_ was evaluated using a catalase assay kit, where the decrease in absorbance at 405 nm reflects H_2_O_2_ decomposition. (**h**) Inhibition rates of H_2_O_2_ decomposition by CeO_2_, Pd + CeO_2_, and Pd@CeO_2_. (**i**) Oxygen generation during H_2_O_2_ decomposition by CeO_2_ NPs, Pd + CeO_2_ NPs, and Pd@CeO_2_

ROS including hydroxyl radicals (·OH), superoxide anions (·O_2_⁻), and hydrogen peroxide (H_2_O_2_), are pivotal in numerous biological and pathological processes [31, 32]. We first evaluated the ·OH scavenging capability of Pd@CeO_2_ using electron spin resonance (ESR) spectroscopy. ESR spectra (Fig. 2a) displayed a distinct 1:2:2:1 quartet signal characteristic of DMPO-·OH adducts generated through the Fenton reaction. Compared to controls, Pd@CeO_2_ exhibited significantly reduced ESR signal intensity, demonstrating superior ·OH scavenging due to the enhanced catalytic effect of Pd@CeO_2_. Subsequently, the SOD-like activity of Pd@CeO_2_ was investigated. ESR spectra of ·O_2_⁻ (Fig. 2c) revealed a pronounced reduction in the typical 1:1:1:1 quartet upon treatment with Pd@CeO_2_, confirming efficient superoxide neutralization. Quantitative analysis using the nitro blue tetrazolium (NBT) assay (Fig. 2d–e) further validated this enhanced performance, as Pd@CeO_2_ exhibited the lowest absorbance at 560 nm, reflecting the highest inhibition rate (48.72%) compared with CeO_2_ (24.81%) and Pd + CeO_2_ mixtures (37.18%). CAT-like activity, essential for H_2_O_2_ detoxification, was evaluated using a colorimetric assay (Fig. 2g–h). Pd@CeO_2_ showed the lowest absorbance at 405 nm and a markedly higher H_2_O_2_ inhibition rate (77.1%) compared to CeO_2_ (40.8%) and Pd + CeO_2_ (44.7%). Additionally, dissolved oxygen measurements revealed rapid catalytic decomposition of H_2_O_2_ to O_2_ upon Pd@CeO_2_ treatment, increasing O_2_ concentrations from 7.5 µg/mL to 28.3 µg/mL within 20 min—significantly outperforming CeO_2_ nanoparticles (21.2 µg/mL) (Fig. 2i). These results demonstrate that Pd@CeO_2_ exhibits comprehensive ROS scavenging capacity, integrating ·OH, ·O_2_⁻, and H_2_O_2_ elimination through synergistic multi-enzyme–like activities.

**Fig. 3:**
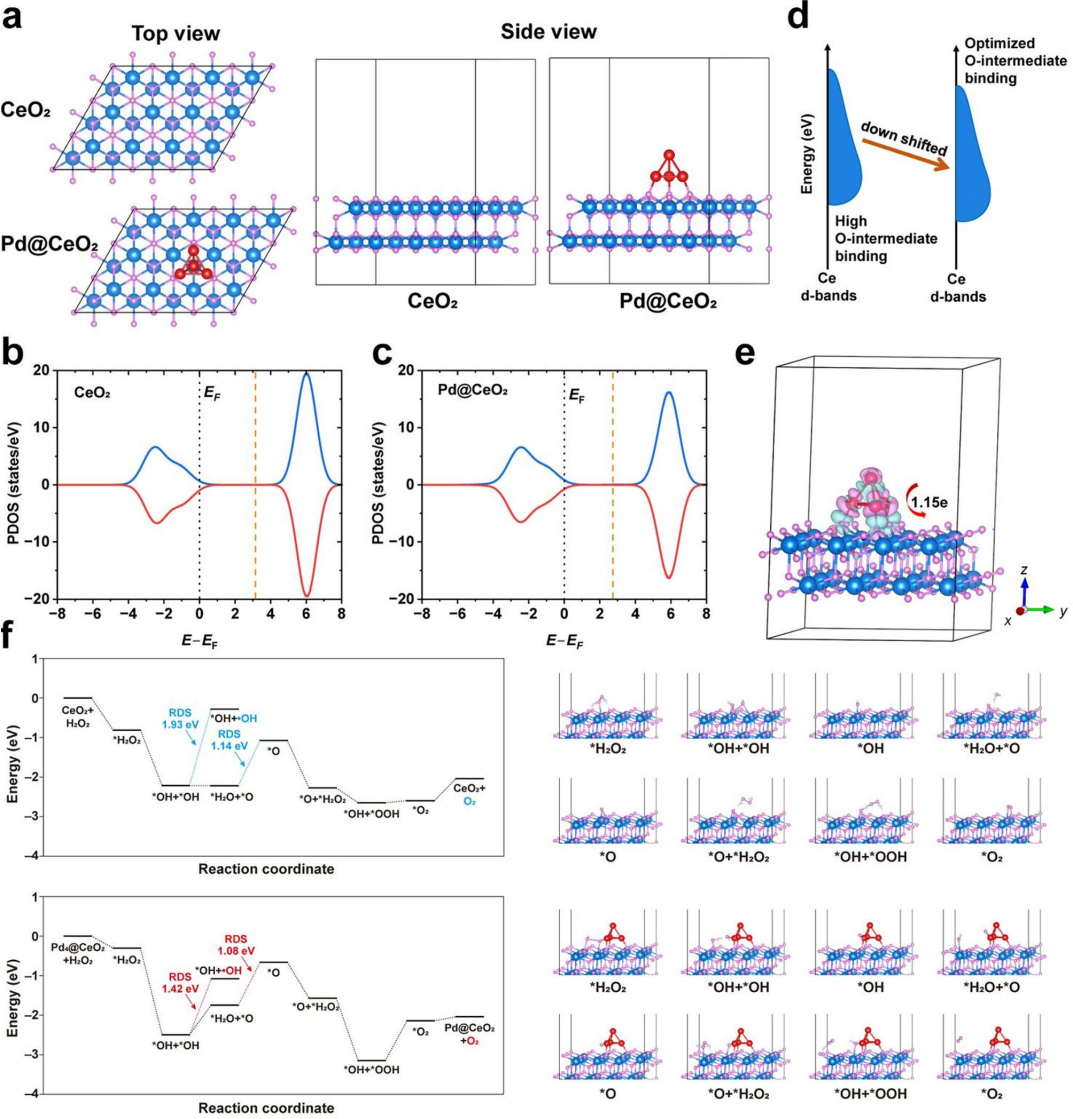
DFT-based mechanistic understanding of Pd@CeO_2_ catalysis. (**a**) Models of the CeO_2_ (111) slab (denoted as CeO_2_) and the CeO_2_ (111) slab with an adsorbed Pd cluster (denoted as Pd@CeO_2_). Spheres in teal, red and grey represent cerium, oxygen and palladium, respectively. (**b**-**c**) PDOS plots of the (**b**) CeO_2_ and (**c**) Pd@CeO_2_. (**d**) Schematic illustration showing that the d-band center shift of CeO_2_ induced by Pd_4_ modulates the binding ability of O-containing intermediates. (**e**) Charge density difference distribution of the Pd@CeO_2_, and insert is Bader charge analysis of Pd_4_ on CeO_2_ (111) slab. The pink and cyan volumes represent electron depletion and accumulation, respectively. Spheres in teal, red and grey represent cerium, oxygen and palladium, respectively. (**f**) Energy profiles and the corresponding configurations for the formation of surface peroxo species on the CeO_2_ (111) slab, either pristine (denoted as CeO_2_) or decorated with a Pd cluster (denoted as Pd@CeO_2_). The rate-determining step (RDS) is highlighted in the plots. Spheres in blue, white, pink and red represent cerium, hydrogen, oxygen and palladium, respectively

To elucidate the mechanism underlying the exceptional catalytic performance of the CeO_2_ (111) slab decorated with Pd clusters, density functional theory (DFT) calculations were performed. A Pd@CeO_2_ model was constructed and compared with the pristine CeO_2_ slab (Fig. 3a). Owing to the pronounced empty-band distribution in CeO_2_, the position of the d-band center relative to the Fermi level was carefully considered. For the CeO_2_ (111) slab, the d-band center is located at 3.13 eV (Fig. 3b). Upon adsorption of a Pd cluster, the d-band center shifts downward by 0.38 eV to 2.75 eV (Fig. 3c), indicating a weakened adsorption strength of oxygen intermediates at the surface Ce sites. These results demonstrate that the incorporation of Pd clusters induces pronounced electronic interactions within the catalyst, resulting in a downward shift of the d-band center. This adjustment optimizes the adsorption of essential intermediates and thereby enhances catalytic efficiency, as illustrated in Fig. 3d. The charge density difference analysis further confirms the electronic modulation induced by the Pd clusters. As shown in Fig. 3e, adsorption of a Pd cluster results in a charge transfer of approximately 1.15 electrons from the cluster to the CeO_2_ substrate, as determined by Bader charge analysis. This electron donation increases the electron density on the substrate, strengthening electronic interactions on CeO_2_. This observation is consistent with the projected density of states (PDOS) analysis, which reveals the downward shift of the d-band center. Furthermore, the adsorption energies of oxygen intermediates on the Pd@CeO_2_ surface were systematically evaluated. The reaction energy barriers of each elementary step were also calculated, revealing a clear relationship between the overlap of transition-metal d orbitals and the adsorption of oxygen intermediates on the catalyst surface (Fig. 3f). For both CeO_2_ and Pd@CeO_2_ systems, we found that the rate-determining step (RDS) for H_2_O_2_ decomposition producing ·OH radicals (i.e., the peroxidase-like reaction) is associated with higher energy barriers compared to H_2_O_2_ decomposition producing O_2_ (i.e., the CAT-like reaction), with values of 1.93 eV vs. 1.14 eV and 1.42 eV vs. 1.08 eV, respectively. This result suggests that the CAT-like pathway predominates in the overall reaction. Notably, the adsorption of Pd clusters significantly reduces the RDS. Specifically, the formation of *O is identified as the RDS for Pd@CeO_2_, with a calculated energy barrier of 1.08 eV, which is lower than the 1.14 eV barrier observed in the pristine CeO_2_ system. This reduction can be attributed to the enhanced electron density at the Ce sites induced by Pd cluster adsorption, which is consistent with the Bader charge analysis, and facilitates H_2_O_2_ reduction and consequently promotes O_2_ evolution, thereby conferring superior CAT-like activity. These findings provide fundamental insights into how Pd clusters regulate the electronic structure of CeO_2_, thereby improving catalytic activity.

### In vitro ROS-scavenging capability of Pd@CeO_2_ nanozymes

**Fig. 4:**
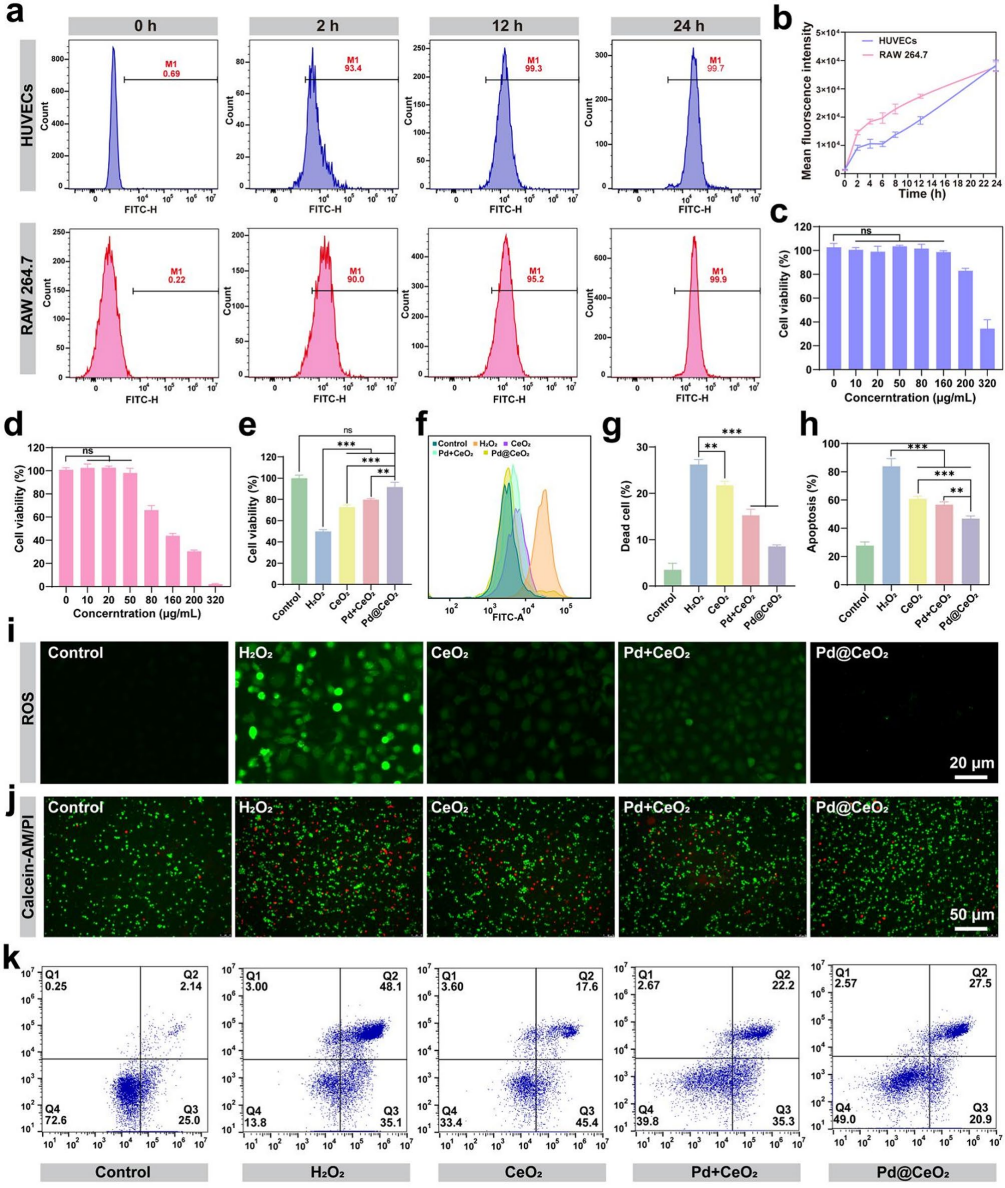
Biocompatibility and protective effects of Pd@CeO_2_ against oxidative damage. (**a**) Flow cytometry analysis of FITC-labeled Pd@CeO_2_ uptake by HUVECs and RAW 264.7 macrophages at different time points (0, 2, 12, and 24 h). (**b**) quantification of flow cytometry results. (**c**) Cytotoxicity of Pd@CeO_2_ on HUVECs at various concentrations (0, 10, 20, 50, 80, 160, 200, and 320 µg/mL). (**d**) Cytotoxicity of Pd@CeO_2_ on RAW 264.7 macrophages at various concentrations (0, 10, 20, 50, 80, 160, 200, and 320 µg/mL). (**e**) Cell viability of CeO_2_, Pd + CeO_2_, and Pd@CeO_2_ (50 µg/mL) against H_2_O_2_-induced oxidative damage. (**f**) Flow cytometric analysis of intracellular ROS levels in oxidatively stressed HUVEC cells. (**g**) Statistical analysis of cell mortality in injured cells. (**h**) Statistical analysis of apoptosis rate in each group. (**i**) DCFH-DA fluorescence microscopy images showing ROS levels in each group. Scale bars: 20 μm. (**j**) Calcein-AM/PI fluorescence staining of HUVECs treated with H_2_O_2_ and or different formulations. Scale bars: 50 μm. (**k**) Flow cytometry analysis of apoptosis rates after different treatments

Building on the robust enzyme-mimetic catalytic activities of Pd@CeO_2_, we evaluated their cellular uptake, biocompatibility, and cytoprotective performance against oxidative stress. The internalization of Pd@CeO_2_ nanozymes was monitored in HUVEC and RAW 264.7 cells using FITC labeling. Fluorescence microscopy and flow cytometry consistently demonstrated efficient, time-dependent cellular uptake of Pd@CeO_2_, with uptake efficiency reaching over 95% within 12 h and approaching complete internalization by 24 h (Fig. 4a–b). Biocompatibility assessment using CCK-8 assays revealed negligible cytotoxicity of Pd@CeO_2_ at concentrations ≤ 50 µg/mL in both HUVEC and RAW 264.7 cells (Fig. 4c–d). Longitudinal analysis over a three-day period confirmed the absence of adverse effects on cell proliferation, highlighting the suitability of Pd@CeO_2_ for biomedical applications (Figure S3). Next, we evaluated the protective role of Pd@CeO_2_ against H_2_O_2_-induced oxidative injury. Pretreatment with Pd@CeO_2_ markedly enhanced HUVEC viability (91.66%), outperforming CeO_2_ (72.72%) and Pd + CeO_2_ mixtures (79.79%) **(**Fig. 4e**)**. Consistently, intracellular ROS levels, visualized by DCFH-DA fluorescence, were substantially diminished in the Pd@CeO_2_ group, underscoring its superior scavenging efficacy relative to the controls **(**Fig. 4f and i and Figure S4–S5). Calcein-AM/PI staining further confirmed enhanced cell survival and reduced cell death following Pd@CeO_2_ treatment under oxidative stress conditions **(**Fig. 4g and j). Flow cytometry analyses using Annexin V-FITC/PI staining demonstrated a substantially lower apoptosis rate in Pd@CeO_2_-treated cells, highlighting robust anti-apoptotic effects (Fig. 4h and k). Collectively, the findings establish Pd@CeO_2_ as a safe and efficient nanozymes with superior antioxidant and anti-apoptotic properties, highlighting its promise for biomedical applications in oxidative stress–driven pathologies.

### Molecular mechanisms of Pd@CeO_2_ in suppressing apoptosis and inflammation

**Fig. 5:**
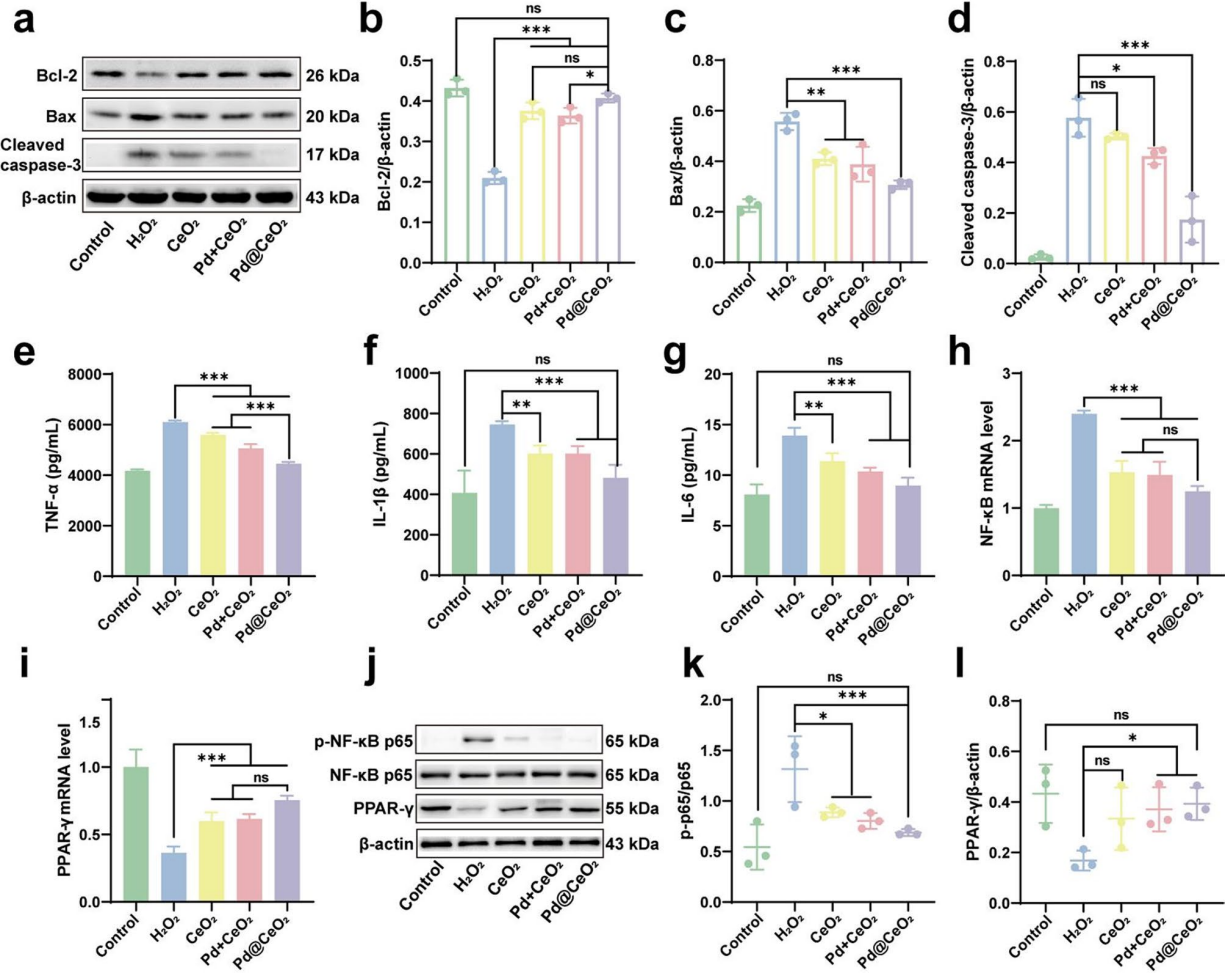
Anti-apoptotic and anti-inflammatory mechanisms of Pd@CeO_2_. (**a**) Western blot analysis of Bcl-2, Bax, and cleaved caspase-3 protein expression levels. Quantification of (**b**) Bcl-2, (**c**) Bax, and (**d**) caspase-3 protein expression levels. ELISA results showing (**e**) TNF-α, (**f**) IL-1β, and (**g**) IL-6 secretion levels after treated with CeO_2_, Pd + CeO_2_, or Pd@CeO_2_. Quantification of (**h**) NF-κB and (**i**) PPAR-γ mRNA expression levels by qPCR. (**j**) Western blot analysis of p-NF-κB p65, NF-κB p65, and PPAR-γ protein expression. Quantification of (**k**) p-p65 and (**l**) PPAR-γ protein expression ratio

To further elucidate the protective mechanisms conferred by Pd@CeO_2_ nanozymes against oxidative stress-induced injury, we evaluated their influence on apoptosis-related pathways. Apoptosis, prominently regulated by proteins including Bcl-2 (anti-apoptotic), Bax (pro-apoptotic), and cleaved Caspase-3 (executioner of apoptosis), is central to I/R injury pathology [33, 34]. Western blot analysis (Fig. 5a–d) demonstrated that Pd@CeO_2_ significantly elevated Bcl-2 expression while concurrently suppressing Bax and cleaved Caspase-3 levels compared to CeO_2_ and Pd + CeO_2_. These findings indicate a robust inhibition of apoptosis via modulation of the Bax/Bcl-2/caspase-3 pathway, underscoring the potent cytoprotective capabilities of Pd@CeO_2_.

Inflammation represents another pivotal aspect of I/R injury, which is further aggravated by excessive ROS generation [35, 36]. Employing an LPS-stimulated RAW 264.7 macrophage inflammation model, ELISA assays revealed that Pd@CeO_2_ markedly attenuated secretion of key pro-inflammatory cytokines TNF-α, IL-1β, and IL-6 compared to the LPS-treated control groups **(**Fig. 5e–g**)**. This highlights the substantial anti-inflammatory efficacy of Pd@CeO_2_ in cellular models. To uncover the molecular basis underlying these anti-inflammatory effects, quantitative PCR and Western blot analyses assessed the expression of inflammation-related factors NF-κB and PPAR-γ. The LPS-driven increase in NF-κB mRNA expression was substantially suppressed by Pd@CeO_2_, highlighting its robust capacity to modulate this pivotal pro-inflammatory transcription factor (Fig. 5h). Conversely, Pd@CeO_2_ significantly upregulated PPAR-γ mRNA, supporting its role in resolving inflammation (Fig. 5i). Protein-level analyses further confirmed these results, demonstrating reduced phosphorylation of NF-κB p65 and increased PPAR-γ protein expression in Pd@CeO_2_-treated groups compared to controls (Fig. 5j–l). These results suggest that Pd@CeO_2_ modulate inflammatory responses by downregulating the NF-κB pathway and upregulating the anti-inflammatory PPAR-γ pathway.

### Restoration of angiogenic capacity by Pd@CeO_2_ in oxidative environments

**Fig. 6:**
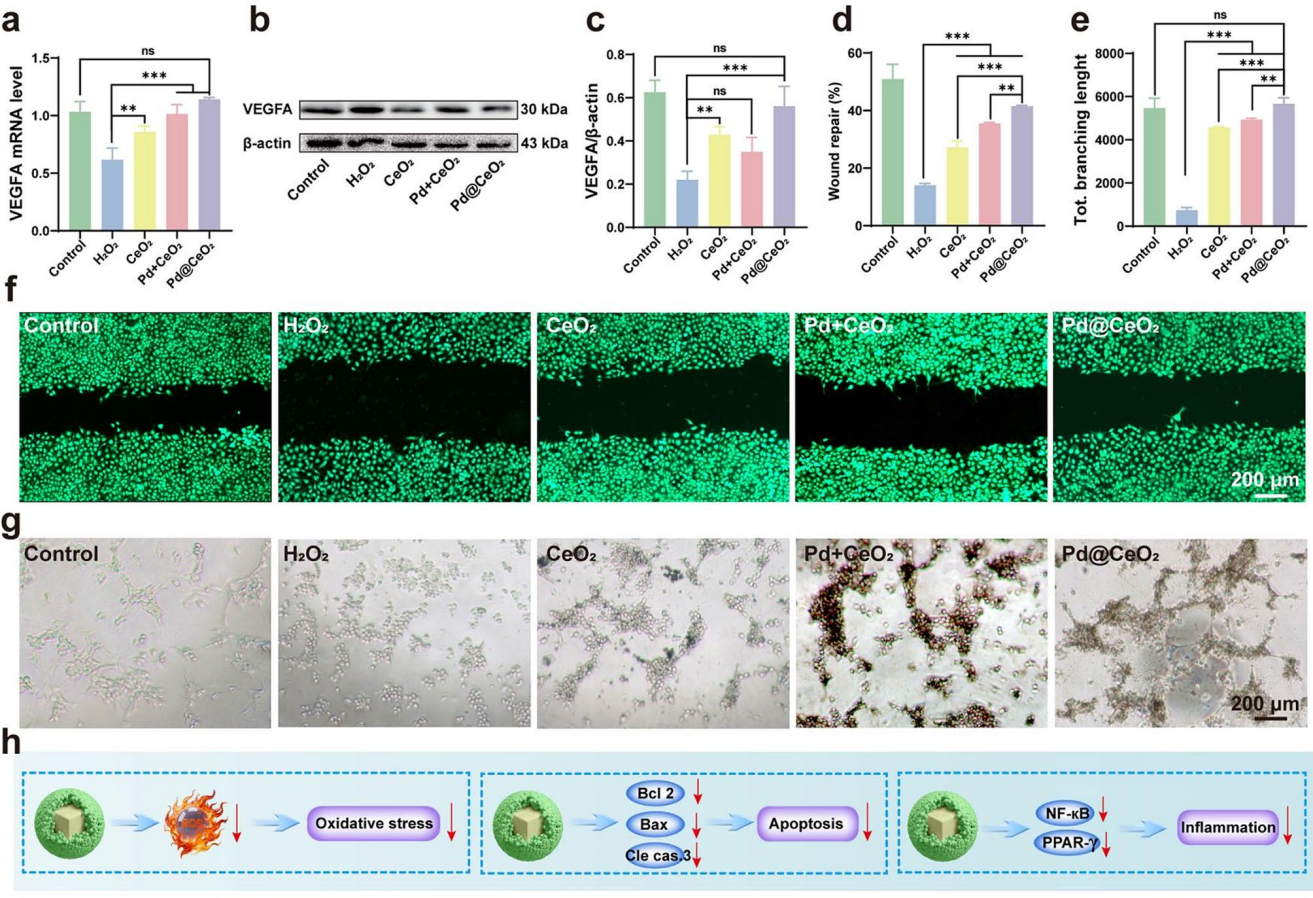
Enhancement of angiogenesis in oxidative stress-damaged HUVECs by Pd@CeO_2_. (**a**) qPCR analysis of VEGFA mRNA expression following treatment with CeO_2_, Pd + CeO_2_, or Pd@CeO_2_. (**b**) Western blot assessment of VEGFA protein expression. (**c**) Quantification of VEGFA protein expression level. (**d**) Statistical analysis of wound repair rate of each group. (**e**) Statistical evaluation of the total dendritic branching length across experimental groups. (**f**) Representative images of the scratch assay in HUVECs under H_2_O_2_-induced oxidative stress. Scale bar: 200 μm. (**g**) Representative images of tube formation assays in HUVECs exposed to H_2_O_2_. Scale bar: 200 μm. (**h**) Diagrammatic representation of Pd@CeO_2_-mediated mechanisms in attenuating oxidative stress, inhibiting apoptosis, and suppressing inflammation

Building upon the demonstrated anti-apoptotic and anti-inflammatory properties of Pd@CeO_2_, we further investigated its angiogenic capacity under oxidative stress, a critical factor in tissue repair and flap survival. Angiogenesis is notably impaired by I/R injury, thus emphasizing its importance in recovery [37, 38]. qPCR analysis revealed that Pd@CeO_2_ treatment markedly upregulated VEGFA mRNA, a key driver of angiogenesis, in H_2_O_2_-stimulated HUVECs compared with controls, CeO_2_, and Pd + CeO_2_ (Fig. 6a). Western blot analyses corroborated this, revealing substantially higher VEGFA protein levels in Pd@CeO_2_-treated cells relative to H_2_O_2_-exposed cells (Fig. 6b–c). These results highlight the strong capacity of Pd@CeO_2_ to reestablish angiogenic signaling pathways disrupted by oxidative stress. We next evaluated endothelial cell migration using scratch assays. H_2_O_2_ exposure markedly impaired migratory capacity, whereas treatment with CeO_2_, Pd + CeO_2_, and especially Pd@CeO_2_ significantly promoted wound closure (Fig. 6d). Experiments further confirmed that Pd@CeO_2_ achieved the most pronounced enhancement of migration, surpassing all other treatments (Fig. 6f). Finally, we assessed angiogenic functionality via endothelial tube formation assays. H_2_O_2_-induced oxidative stress severely disrupted tube formation, evident by reduced branching and network complexity. Notably, pretreatment with Pd@CeO_2_ effectively restored endothelial tube formation, significantly enhancing total branching length compared to CeO_2_ and Pd + CeO_2_ treatments ((Fig. 6e and g). These collective results position Pd@CeO_2_ as a highly effective nanozyme, capable of significantly promoting angiogenesis under oxidative stress, thereby highlighting its substantial therapeutic potential in ischemic tissue repair.

In vitro investigations revealed that Pd@CeO_2_ orchestrates multifaceted cytoprotective mechanisms by reducing oxidative stress, inhibiting apoptosis through the Bcl-2/Bax/caspase axis, and dampening inflammatory cascades mediated by NF-κB and PPAR-γ (Fig. 6h), thereby establishing a coherent mechanistic basis for its therapeutic potential.

### Therapeutic efficacy of Pd@CeO_2_ in a rat skin flap ischemia–reperfusion injury model

To preliminarily assess the in vivo biosafety of Pd@CeO_2_ nanozymes, we examined their biocompatibility through hemocompatibility and histological studies. Hemolysis assays revealed an exceptionally low hemolysis rate (< 10%) across concentrations up to 50 µg/mL, indicating excellent blood compatibility (Figure S6). Blood routine and biochemical analyses performed 14 days post-injection revealed that major hematological indices, as well as hepatic and renal function markers (ALT, AST, BUN, and creatinine), remained within physiological ranges and were indistinguishable from those of the control group, thereby substantiating the systemic biosafety of the treatment (Table S1-S2). Histological examination of major organs (heart, liver, spleen, lung, and kidney) by H&E staining at 7 and 14 days revealed no detectable pathological alterations, thereby reinforcing the excellent biosafety profile of Pd@CeO_2_ (Figure S7).

**Fig. 7:**
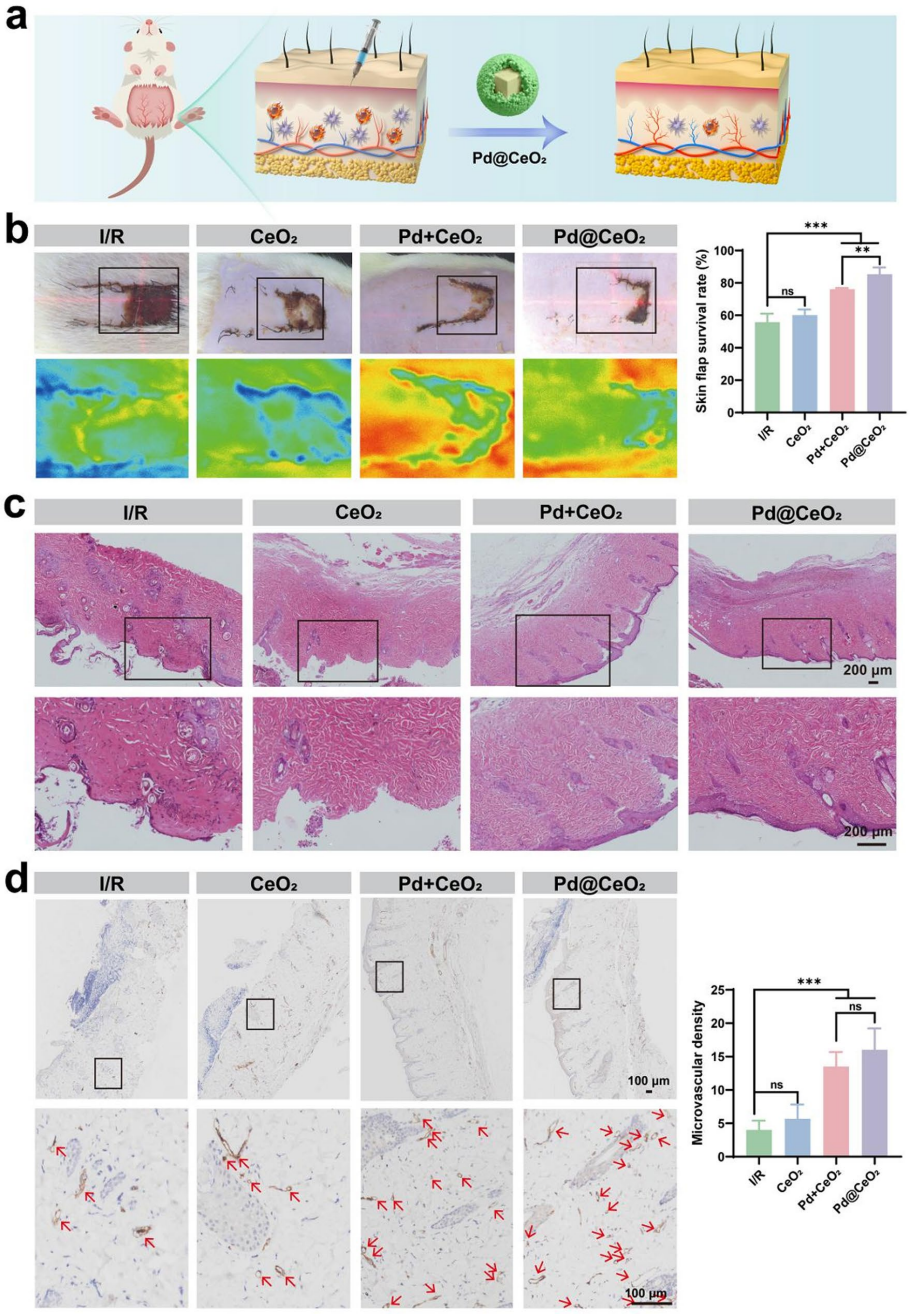
Evaluation of the therapeutic effects of Pd@CeO_2_ in a rat skin flap I/R injury model. (**a**) Schematic illustration of the therapeutic process in the I/R injury model. (**b**) Representative images of skin flaps on day 7 post-surgery for the I/R, CeO_2_, Pd + CeO_2_, and Pd@CeO_2_. (**c**) HE staining of distal skin flap tissue sections. Scale bars: 200 μm. (**d**) Representative images of immunohistochemical staining of CD31 in I/R, CeO_2_, Pd + CeO_2_, and Pd@CeO_2_ groups

Subsequently, therapeutic efficacy was evaluated using a rat dorsal skin flap I/R injury model. After 6 h of ischemia followed by reperfusion, extensive flap necrosis was observed in the untreated controls, whereas Pd@CeO_2_ treatment markedly preserved tissue integrity and minimized necrotic damage (Fig. 7a-b). Real-time microcirculation assessed via laser speckle imaging revealed significantly enhanced blood perfusion in the Pd@CeO_2_ group, which achieved the highest flap survival rate (85.42%) compared to CeO_2_ (60.10%), Pd + CeO_2_ (77.14%), and untreated groups (55.76%) (Fig. 7b). H&E staining further demonstrated significant preservation of tissue architecture in Pd@CeO_2_-treated flaps, characterized by intact epidermal layers, well-maintained hair follicles, and normal dermal collagen deposition, contrasting sharply with severe epidermal thinning in control groups (Fig. 7c). Given the promising in vitro angiogenic effects of Pd@CeO_2_, we subsequently evaluated its pro-angiogenic capabilities within an in vivo I/R skin flap model. Immunohistochemical staining for the endothelial marker CD31 was performed to assess neovascularization within flap tissues. Results indicated compromised angiogenesis in the I/R group, exhibiting microvascular densities of approximately 4.0 vessels per field. In contrast, groups treated with Pd + CeO_2_ and particularly Pd@CeO_2_ displayed considerably enhanced neovascularization, with microvessel densities of 13.5 and 16.0, respectively (Fig. 7d). These findings underscore the superior efficacy of Pd@CeO_2_ in promoting angiogenesis under I/R injury conditions.

### Mitigation of inflammation and apoptosis by Pd@CeO_2_ in I/R skin flaps

**Fig. 8:**
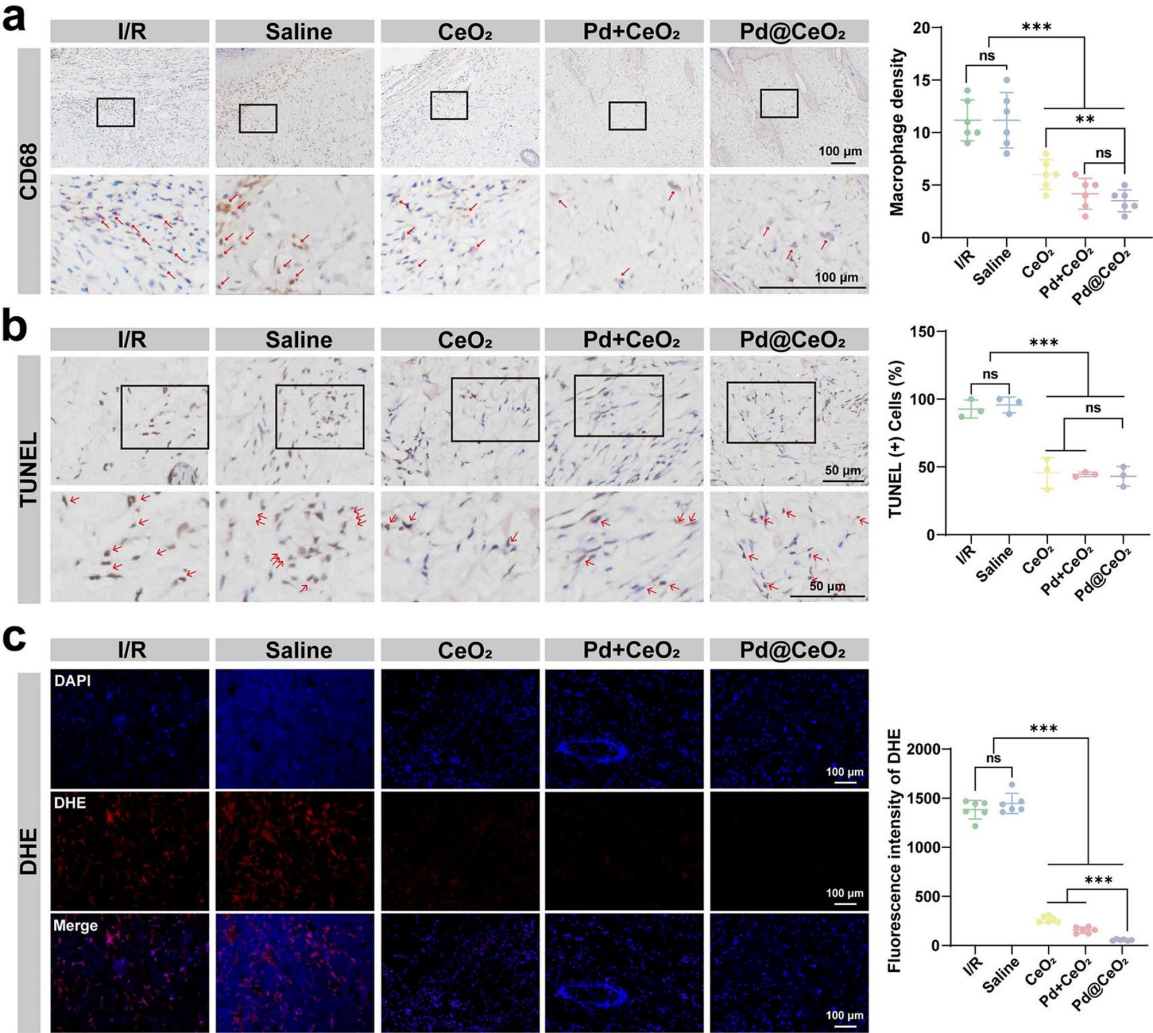
Anti-inflammatory and anti-apoptotic effects of Pd@CeO_2_ in an I/R skin-flap model. (**a**) Immunohistochemical staining and semi-quantitative analysis of CD68 to evaluate macrophage densities in the skin flap tissues, with red arrows indicating macrophages, bar = 100 μm. (**b**) TUNEL staining and quantitative assessment to detect apoptotic cells in skin flap tissues, with red arrows highlighting apoptotic cells. Scale bars: 50 μm. (**c**) ROS levels and semi-quantitative analysis in skin flap tissues assessed via DHE staining. Scale bars: 100 μm

To further validate the anti-inflammatory potential of Pd@CeO_2_, immunohistochemical staining of CD68 was conducted to quantify macrophage infiltration, indicative of inflammation within skin flaps. As shown in Fig. 8a, markedly elevated macrophage densities were detected in both the I/R and saline groups (≈ 11.17 each), reflecting pronounced inflammatory activation. In contrast, macrophage infiltration was substantially reduced in the CeO_2_ (≈ 6.0) and Pd + CeO_2_ groups (≈ 4.17), with the most pronounced attenuation observed in the Pd@CeO_2_-treated group (≈ 3.5), thereby underscoring the potent in vivo anti-inflammatory efficacy of Pd@CeO_2_. Additionally, TUNEL assays were employed to evaluate apoptosis within the flap tissues. Distinctly higher densities of TUNEL-positive cells were detected in the I/R and saline control groups, reflecting significant apoptotic activity (apoptosis rates of 92.67% and 95.67%, respectively). Treatment groups showed substantially reduced apoptosis rates, with CeO_2_ at 45.68%, Pd + CeO_2_ at 44.49%, and notably, Pd@CeO_2_ demonstrating the lowest apoptosis at 43.08% (Fig. 8b). Finally, DHE staining indicated that Pd@CeO_2_ treatment effectively suppressed ROS accumulation in flap tissues, reinforcing the correlation between robust ROS scavenging and improved flap viability and structural integrity (Fig. 8c). These results collectively confirm that Pd@CeO_2_ effectively mitigates apoptosis, potentially via robust ROS scavenging and reduced inflammatory signaling, thus underscoring its substantial therapeutic potential in addressing oxidative and inflammatory damage during I/R injury.

### Transcriptomic analysis of Pd@CeO_2_-mediated protection against I/R injury

**Fig. 9:**
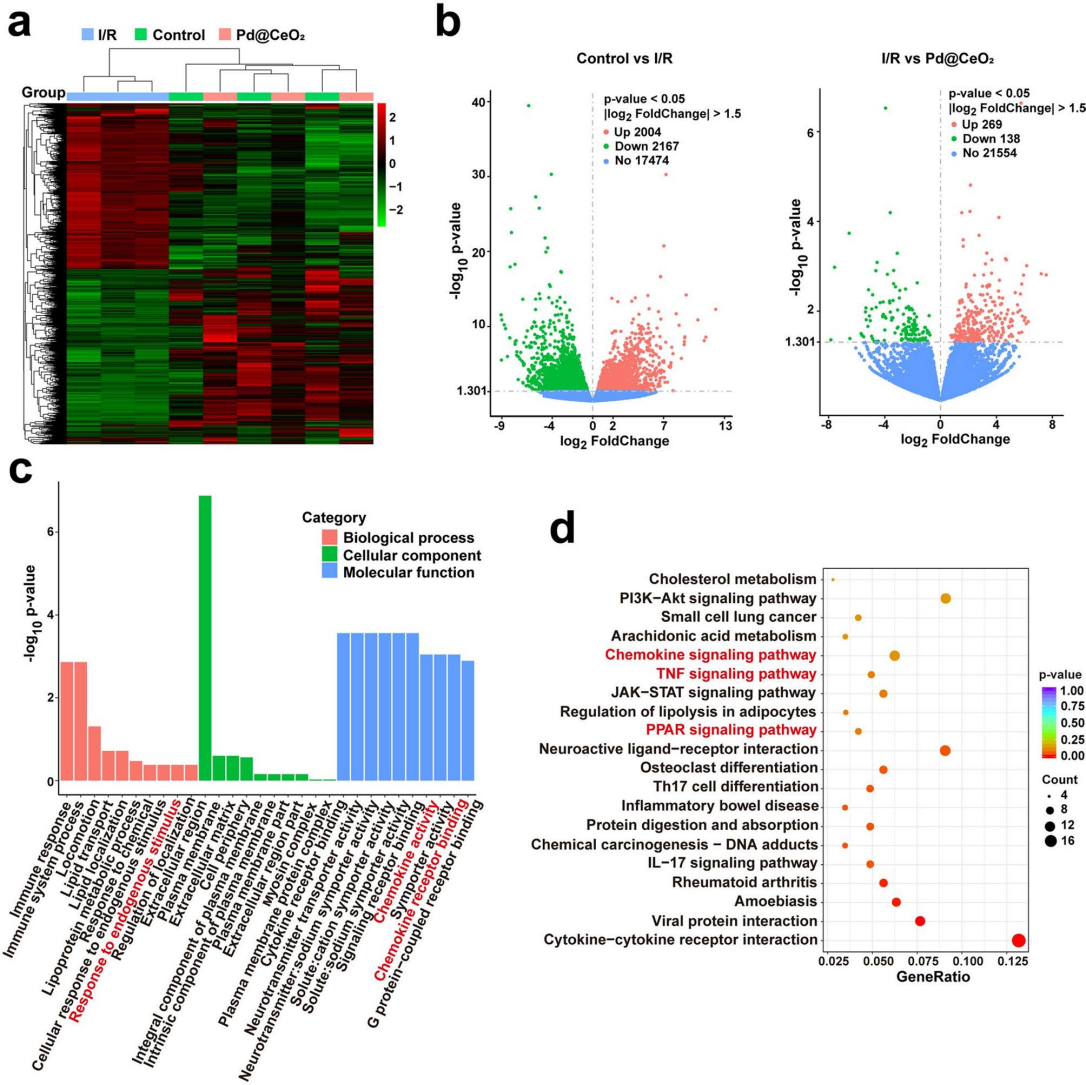
In vivo transcriptomic analysis of rat with I/R injury. (**a**) Hierarchical clustering heatmap depicting DEG in different groups. **(b**) Volcano plots. (**c**) GO enrichment analysis. (**d**) KEGG pathway enrichment scatterplot

To gain comprehensive insights into the molecular mechanisms underlying the therapeutic actions of Pd@CeO_2_ in I/R injury, transcriptomic profiling was conducted. Hierarchical clustering of differentially expressed genes (DEGs) revealed distinct transcriptional signatures among the Control, I/R, and Pd@CeO_2_-treated groups (Fig. 9a). Volcano plots further delineated significantly altered gene expressions, highlighting 2004 upregulated and 2167 downregulated genes when comparing the control and I/R groups. In contrast, Pd@CeO_2_ treatment induced significant expression changes, with 269 genes upregulated and 138 genes downregulated compared to the I/R group (Fig. 9b). Gene Ontology (GO) enrichment analysis indicated that Pd@CeO_2_ treatment notably influenced biological processes related to inflammation and immune responses, cellular components associated with membrane and extracellular matrix, and molecular functions involving receptor activity and signaling transduction (Fig. 9c). Notably, enriched biological processes such as “inflammatory response”, “immune response”, and “cellular response to cytokine stimulus” were significantly modulated upon Pd@CeO_2_ treatment, indicating its robust anti-inflammatory potential at the transcriptomic level. Kyoto Encyclopedia of Genes and Genomes (KEGG) pathway analysis further highlighted key signaling pathways significantly regulated by Pd@CeO_2_ treatment, including TNF, Chemokine, and PPAR signaling pathways (Fig. 9d). These results corroborate earlier findings regarding the involvement of these pathways in inflammatory modulation and tissue repair, providing robust transcriptomic validation for the anti-inflammatory and regenerative effects observed in previous cellular and histological assessments.

## Conclusion

In summary, we have successfully developed a novel Pd@CeO_2_ nanozyme characterized by a unique core-shell structure exhibiting SOD and CAT-like activities. Our comprehensive results demonstrate that Pd@CeO_2_ effectively scavenges ROS, significantly enhances cell viability, and promotes angiogenesis under I/R conditions. Mechanistically, Pd@CeO_2_ mitigates oxidative stress-induced apoptosis through modulation of the Bax/Bcl-2/caspase-3 signaling pathway, effectively attenuates inflammation via the PPAR-γ/NF-κB axis, and substantially restores vascularization through enhanced VEGFA expression. In vivo studies using a rat skin flap I/R injury model validated these findings, where Pd@CeO_2_ markedly improved flap survival, reduced tissue inflammation and apoptosis, and enhanced microvascular density. Additionally, rigorous biocompatibility evaluations confirmed the high safety profile of Pd@CeO_2_, characterized by minimal hemolytic activity, stable blood biochemistry, and absence of detectable organ toxicity. The designed Pd@CeO_2_ offers a versatile strategy for clinical interventions in ischemic tissue injuries and advancing the broader field of regenerative medicine.

### Supporting information

Supporting information is available from the Journal of Nanobiotechnology or from the author.

## Supplementary Information

Supplementary Material 1

## Data Availability

No datasets were generated or analysed during the current study.
