# Supplementary material for: Electron-injected Pd@CeO2 nanozymes for multifaceted ROS scavenging and protection against ischemia-reperfusion injury in skin flaps
Source: J Nanobiotechnology. 2025 Oct 21;23:686. doi: 10.1186/s12951-025-03775-3 (PMC12538877; doi:10.1186/s12951-025-03775-3)
Supplement: Supplementary file 1 — Supplementary Material 1 [file 12951_2025_3775_MOESM1_ESM.docx]

**Supporting information**

**Electron-injected Pd@CeO_2_ nanozymes for multifaceted ROS scavenging and protection against ischemia-reperfusion injury in skin flaps**

*Lingling Zhou^1,2^**^†^, Jiayuan Sun^5†^, Tianxiang Lu^4†^, Xinya Zhang^7^,* *Mingkang Wang^6^, Yanjun Xu^8^, Jia Zhou^1,2^, Xiaoyang Li^9^, Wenxian Du^1,2^*, Fan Yang^3^*, Yuehua Li^1,2^**

1 Department of Radiology, Shanghai Jiao Tong University School of Medicine Affiliated Sixth People’s Hospital, Shanghai, 200233, People’s Republic of China;

2 Faculty of Medical Imaging Technology, College of Health Science and Technology, Shanghai Jiao Tong University School of Medicine

3 Department of Pharmacy, Renji Hospital, School of Medicine, Shanghai Jiao Tong University, Shanghai, 200127, People’s Republic of China;

4 Department of Obstetrics and Gynecology, Xijing Hospital Affiliated to the Fourth Military Medical University, Xian, 710032, People’s Republic of China;

5 Department of Plastic and Reconstructive Surgery, Ninth People’s Hospital Affiliated to Shanghai Jiao Tong University School of Medicine, Shanghai, 200011, People’s Republic of China;

6 Wuhan United Imaging Life Science Instruments Ltd., Wuhan, 430000, People’s Republic of China;

7 Jiangsu Health Vocational College, Nanjing, 210000, People’s Republic of China;

8. Department of Ultrasound in Medicine, Shanghai Sixth People's Hospital Affiliated to Shanghai Jiao Tong University School of Medicine, Shanghai, 200233, People’s Republic of China;

9 School of Agriculture and Biology, Shanghai Jiao Tong University, 800 Dongchuan Road, Shanghai, 200240, People’s Republic of China;

**Material**

Sodium tetrachloropalladate (Na_2_PdCl_4_), L-ascorbic acid (AA), polyvinylpyrrolidone (PVP), potassium bromide (KBr), cerium nitrate, and L-arginine were purchased from Sigma-Aldrich. Acetone and ethanol were obtained from China National Pharmaceutical Group Corporation (Sinopharm). DMPO was supplied by Radical Vision Co., Ltd.. SOD activity assay kit and CAT activity detection kit were purchased from Boxbio Biotechnology Co., Ltd.. High-glucose DMEM medium was sourced from Hyclone Laboratories. Fetal bovine serum (FBS) and 0.25% trypsin-EDTA solution were obtained from Thermo Fisher Scientific, Gibco™. All chemicals and reagents were used as received without further purification.

**Preparation of Pd Cubes**

60 mg of AA, 105 mg of PVP (MW ≈ 55,000), and 600 mg of KBr were added to 8 mL of water. After fully dissolved, the mixture was then heated at 80°C for 10 min. Subsequently, 3.029 mL of Na_2_PdCl_4_ solution (0.0640 M) was rapidly injected into the reaction mixture. After 3 h, the product was precipitated using acetone and collected via centrifugation. The purified palladium cubes were resuspended in 20 mL of water for further use.

**Synthesis of Pd@CeO_2_**

0.2 mmol of Ce(NO_3_)_3_ was dissolved in 20 mL of water and 20 mL of ethanol. Next, 5 mL of the prepared Pd cubes solution was added. While stirring at room temperature, 10 mL of L-arginine solution (0.085 g in 10 mL of water) was gradually added dropwise. The mixture was then heated at 80°C for 3 h. The product was collected via centrifugation with the aid of acetone and redispersed in 10 mL of water for further use.

**Characterization**

TEM imaging was performed on a JEM-2100 microscope, while XRD measurements were carried out using a DMAX-2400 diffractometer. XPS analysis was conducted with an ESCALAB 250 system, and ESR spectra were recorded on a Bruker EMX1598 spectrometer.

**ROS scavenging assay**

**·OH scavenging assay**: DMPO (5 μL, 10 M) was dissolved in 5 mL of PBS (10 mM), and Fe²⁺ (20 μL, 50 mM) was added to catalyze the decomposition of H₂O₂ to produce •OH radicals.

**SOD-like activity assay**: SOD activity assay kit was used where xanthine reacts with xanthine oxidase to generate ·O_2_⁻, which reduce nitro blue tetrazolium (NBT) to form a blue formazan product. Additionally, ESR spectroscopy was used to evaluate SOD activity. ·O_2_⁻ were generated by dissolving 70 μg of KO_2_ with 200 μL of 18-crown-6 in DMSO (0.7 mM).

**CAT-like activity assay**: Nanozymes with CAT-like properties decompose H_2_O_2_, while the remaining H_2_O_2_ oxidizes TiSO_4_ to produce yellow titanium peroxysulfate. Additionally, the oxygen generation capacity in the presence of H_2_O_2_ was evaluated using a dissolved oxygen meter.

**Cell viability assessment**
The cytotoxicity of Pd@CeO_2_ was evaluated using HUVEC and RAW 264.7 cell lines. Cells were seeded into 96-well plates (1×10^4^ cells per well). After overnight incubation, 200 μL of culture medium with or without different formulations were added. The cells were then cultured at 37°C in a 5% CO_2_ incubator for 24 h. After treatment, the wells were washed three times with PBS, and 100 μL of serum-free DMEM medium containing 10% CCK-8 reagent was added to each well. Plates were incubated for 2 h in the dark, and absorbance at 450 nm was subsequently measured with a microplate reader.

**Cell proliferation assessment**
Cells were seeded into 96-well plates (1×10^3^ cells per well). After cell adhesion, 200 μL of culture medium with or without nanozyme was added. The cells were cultured at 37°C in a 5% CO₂ incubator for 24–72 h. At 24, 48, and 72 h, the cell number was measured using the CCK-8 assay described above. Cell proliferation rates were calculated accordingly.

**Preparation of FITC-labeled Pd@CeO_2_**
FITC-labeled Pd@CeO_2_ were prepared by dissolving 1 mg of FITC in DMSO and adding it to the Pd@CeO_2_ solution. The mixture was stirred overnight under dark conditions using a magnetic stirrer. The resulting product was centrifuged and washed three times with water and ethanol, respectively.

**Cell culture and incubation**
HUVEC and RAW 264.7 cells were cultured in 24-well plates following standard procedures. Once the cell confluency exceeded 80%, the culture medium was replaced with fresh complete medium containing FITC-labeled Pd@CeO_2_ at a concentration of 50 μg/mL. After 2 h of incubation, intracellular fluorescence was observed and imaged using a fluorescence microscope.

**Time-dependent uptake analysis**
To assess the time-dependent uptake, cells were co-incubated with FITC-labeled Pd@CeO_2_ for 2 h, 4 h, 6 h, 8 h, 12 h, and 24 h. At each time point, cells were collected under dark conditions. The cells were transferred into EP tubes and centrifuged at room temperature (1000 rpm, 10 min) to collect the cell pellets. The pellets were resuspended in 300–500 µL of PBS and measured using a flow cytometer at a wavelength of 525 nm.

**Intracellular ROS scavenging by Pd@CeO_2_**

The intracellular ROS levels were evaluated using DCFH-DA. HUVECs were seeded in six-well plates and allowed to adhere, followed by treatment with H_2_O_2_ at a final concentration of 500 μM after 2 h of incubation. Cells cultured with or without different formulations. After a 2-h incubation, the cells were washed three times with PBS, and the medium was replaced with serum-free medium containing DCFH-DA (1:1000 dilution). The cells were incubated in the dark for 30 min, followed by washing three times with serum-free medium to remove excess DCFH-DA. Intracellular ROS levels were observed under a fluorescence microscope. Additionally, cells were collected, centrifuged at 1000 rpm for 10 min, and resuspended in PBS. The fluorescence intensity was quantified using flow cytometry at an excitation wavelength of 488 nm and emission wavelength of 525 nm.

**Computational methodologies**

Density functional theory (DFT) calculations were performed by using the QUANTUM ESPRESSO package ^[1,2]^. The exchange-correlation functional was approximated within the framework of the generalized gradient approximation (GGA), specifically employing the Perdew-Burke-Ernzerhof (PBE) parametrization ^[3]^. Ionic potentials were described by means of ultrasoft pseudopotentials (USPPs), which were retrieved from the official Quantum-ESPRESSO website. All calculations were performed with spin polarization included. The energy cutoff for the wave functions and for the charge density were equal to 50 Ry and 350 Ry, respectively. The Brillouin zone was sampled using a k-point grid of 3×2×1 points. The convergence threshold was set to be 10^-5^ Ry for the total electronic energy in the self-consistent loop. For the CeO_2_ (111) surfaces, an infinite slab model consisting of six atomic layers was adopted. A Pd cluster containing four Pd atoms was adsorbed onto the (111) surface facets. All slabs were separated by a vacuum region exceeding 15 Å along the z-direction to avoid spurious interactions between periodic images. During the geometry optimization process, the bottom three atomic layers were kept rigid to simulate the bulk structure, while the top three atomic layers of the CeO_2_ (111) surface, along with the Pd cluster and all adsorbates, were allowed to fully relax.

**CCK-8 assay for cell protection by Pd@CeO_2_**

HUVECs were seeded into 96-well plates (1×10^4^ cells per well). After adherence, the medium was replaced with different medium. After 2 h of incubation, H_2_O_2_ was added to a final concentration of 500 μM. After treatment, cell viability was measured using the CCK-8 assay, following the procedure and calculation method described earlier.

**Live/dead staining**

Cells were seeded into 6-well plates, and the treatment groups were set up as described above. After stimulation with 500 μM H_2_O_2_ for 2 h, all cells were collected and resuspended in AM/PI staining solution. The cells were incubated in the dark at 37°C for 30 min. After incubation, cells were centrifuged, washed, and resuspended in 100 μL of buffer. The cell suspension was placed on slides and observed under a confocal microscope.

**Apoptosis detection by Annexin-FITC/PI staining**

The sample preparation, treatment groups, and procedure were the same as described above. Both the culture medium and digested cells were collected, centrifuged and washed. The cell pellet was resuspended in 210 μL of working solution containing 5 μL Annexin-FITC and 10 μL PI. The samples were incubated at room temperature in the dark for 15 min, with gentle inversion every 5 min. After staining, cells were washed, resuspended, and dispersed. Flow cytometry analysis was performed within 1 h using an excitation/emission wavelength of 488/525 nm for FITC and 535/615 nm for PI.

**Western blot analysis of apoptosis pathways**

**Protein extraction:** HUVECs were seeded in 6-well plates, and oxidative damage was induced as described earlier. Cells were washed twice with PBS, and 200 μL of RIPA lysis buffer containing protease inhibitors was added to each well. The plates were kept on ice for 15–30 min, and the cells were scraped to ensure complete lysis. The lysate was centrifuged and the supernatant was collected, and protein concentration was determined using the BCA assay.

**BCA protein assay:** BCA working solution was prepared by mixing 6 mL of reagent A with 120 μL of reagent B. Standards were prepared by diluting a 5 mg/mL stock to final concentrations ranging from 0 to 0.5 mg/mL. Standards and samples were added to a 96-well plate in duplicate, followed by the addition of BCA working solution. The plate was incubated at 37°C for 30 min, and absorbance was measured at 570 nm.

**Western Blot:** Proteins were denatured at 100°C for 10 min and loaded onto an SDS-PAGE gel. Electrophoresis was performed, and proteins were transferred to a PVDF membrane. The membrane was blocked with milk, incubated with primary antibodies overnight, and then with secondary antibodies for 1 h. After washing, the membrane was developed using an ECL detection kit, and the bands were visualized. Image J software was used for analysis.

**Anti-inflammatory effect of Pd@CeO_2_ via ELISA**

RAW 264.7 cells were seeded into 6-well plates (2×10^5^ cells per well). After adhesion, cells were co-cultured with the indicated groups for 2 h and subsequently stimulated with LPS at a final concentration of 500 ng/mL, while cells cultured without LPS served as controls. After 24 h of incubation, the supernatants were collected and centrifuged to remove cellular debris, followed by quantification of inflammatory cytokines using ELISA kits in accordance with the manufacturer’s instructions.

**RT-PCR for inflammation pathway analysis**

Total RNA was extracted from RAW 264.7 cells using the Trizol method. Total RNA was reverse-transcribed into cDNA, and gene expression was quantified by qPCR. The reaction was performed in a 20 µL system, including 10 µL PCR Premix, 10 ng cDNA template, and 0.2 µL of each primer. Amplification was conducted with the following cycles: 95°C for 5 min, followed by 40 cycles of 95°C for 15 s and annealing at 55–60°C. Primer sequences were as follows:

- **NF-κB p65**:
  - Sense: 5'-TGCAGAAAGAAGACATTGAGGTG-3'
  - Antisense: 5'-AGGCTAGGGTCAGCGTATGG-3'
- **PPAR-γ**:
  - Sense: 5'-GCGGAGATCTCCAGTGATATC-3'
  - Antisense: 5'-TCAGCGACTGGGACTTTTCT-3'.

**Cell migration assay**

Cells were seeded in 24-well plates, with treatment groups established as previously described. After 2 h of treatment with 500 μM H_2_O_2_, a straight scratch was made at the bottom of the well using a 200 μL pipette tip. The wells were washed three times with PBS to remove detached cells, followed by the addition of fresh serum-free medium. The scratches were observed and photographed under an inverted microscope. The plates were incubated under standard culture conditions, and after 24 h, cells were stained with Calcein AM for 30 min. Fluorescence images were captured, and scratch areas were measured using Image J software.

**Tube formation assay**

Cells were collected and seeded into 12-well plates following the grouping and treatment protocols described previously. After treatment, cells were harvested, counted, and resuspended in serum-free DMEM. The resuspended cells (1×10^4^ cells per well) were seeded onto Matrigel-coated 96-well plates. Tube formation was examined and imaged using an inverted microscope.

**Ischemia-reperfusion (I/R) skin flap model**

Rats were anesthetized with 2% sodium pentobarbital (40 mg/kg, intraperitoneal injection). Hair was removed from the dorsal region using a shaver and depilatory cream. A 1.5×6 cm rectangular skin flap, parallel to the axial line, was designed on the back with the pedicle along the bilateral iliac crest line. After sterilization with povidone-iodine, the skin was incised along the marked line to the superficial fascia using a sterile scalpel. The bilateral sacral arteries at the pedicle base were ligated, and the distal half of the subcutaneous tissue within the flap was excised. The skin flap was sutured back in place with 5-0 sutures to establish the I/R injury model. After 7 days, the rats were re-anesthetized, and the representative blood flow at the distal junction of the flap was detected using laser speckle imaging. Rats were divided into five groups: I/R group (surgery only), I/R+saline group (intradermal injection of 1 mL saline 2 h pre-surgery), I/R+CeO_2_ group (intradermal injection of 50 μg/mL CeO_2_, 1 mL, 2 h pre-surgery), I/R+Pd+CeO_2_ NPs group (intradermal injection of 50 μg/mL Pd+CeO_2_, 1 mL, 2 h pre-surgery), and I/R+Pd@CeO_2_ group (intradermal injection of 50 μg/mL Pd@CeO_2_, 1 mL, 2 h pre-surgery).

**H&E staining**

On postoperative day 7, rats were euthanized with an overdose of chloral hydrate, and tissue samples (0.5 × 2 cm) from the flap center were harvested and fixed in 4% paraformaldehyde at 4°C overnight. The specimens were subsequently dehydrated through a graded ethanol series, cleared in xylene, and embedded in paraffin. Paraffin blocks were sectioned into 5-μm slices using a microtome, mounted onto glass slides, and heat-dried. The slides were deparaffinized in xylene, rehydrated through descending ethanol concentrations, and stained sequentially with hematoxylin (5 min) and eosin (3 min). After dehydration and neutral resin mounting, histological images were acquired under a light microscope.

**Dihydroethidium (DHE) staining**

Prior to staining, slides were pre-warmed and incubated with DHE diluted in PBS at 37°C for 30 min in the dark. Following three washes with PBS, nuclei were counterstained with DAPI for 10 min. The slides were then rinsed, air-dried, and mounted using an antifade medium. Fluorescent signals were subsequently visualized and imaged with an inverted fluorescence microscope.

**Immunohistochemical staining**

Paraffin-embedded sections were deparaffinized in xylene twice (10 min each) and rehydrated through a graded ethanol series. Antigen retrieval was carried out in 0.01 M EDTA buffer (pH 9.0) under high temperature and pressure. Endogenous peroxidase activity was quenched with 3% H_2_O_2_, followed by PBS rinses. The sections were then incubated with primary antibodies (CD31, 1:1000; CD68, 1:500) at 37°C for 1 h. After washing, HRP-conjugated secondary antibodies were applied at 37°C for 30 min. Color development was achieved using DAB substrate for 1 min, and the reaction was terminated by rinsing in water. Sections were subsequently counterstained with hematoxylin, dehydrated, cleared in xylene, and mounted. Stained slides were scanned under a light microscope, and three randomly selected fields per section were quantitatively analyzed using ImageJ software.

**TUNEL staining of flap tissues**

Paraffin-embedded sections were deparaffinized and rehydrated through a graded ethanol series, followed by rinsing in deionized water. Endogenous peroxidase activity was quenched with 0.3% H_2_O_2_ in methanol for 10 min and washed with PBS. Sections were then digested with proteinase K at 37°C for 10 min, rinsed in PBS, and pretreated with the kit-provided buffer on ice for 2 min. The TUNEL reaction mixture was subsequently applied, and slides were incubated at 37°C for 1 h. After PBS washing, HRP-conjugated secondary antibodies were applied and incubated at 37°C for 30 min. Color development was performed using DAB, with the reaction monitored microscopically. Nuclei were counterstained with hematoxylin, and sections were dehydrated, cleared, and mounted. Apoptotic nuclei appeared brown, whereas non-apoptotic nuclei were stained blue under light microscopy.

**RNA sequencing (RNA-seq)**

Brains were harvested from the control, I/R, and Pd@CeO_2_ groups (n = 3 per group). Following anesthesia and transcardial perfusion, brain tissues were rapidly excised, snap-frozen in liquid nitrogen, and stored at –80 °C until RNA extraction. RNA integrity was assessed using the RNA Nano 6000 Assay Kit, and only high-quality samples were used for library construction. Differential expression analysis was performed with DESeq2, applying thresholds of FDR < 0.05 and |log2FC| > 1. Functional enrichment of differentially expressed genes was conducted using GO and KEGG pathway enrichment analysis.

**References：**

[1] G.L. Chiarotti, M. Cococcioni, I. Dabo, A. Dal Corso, S. de Gironcoli, S. Fabris, G. Fratesi, R. Gebauer, U. Gerstmann, C. Gougoussis, A. Kokalj, M. Lazzeri, L. Martin-Samos, N. Marzari, F. Mauri, R. Mazzarello, S. Paolini, A. Pasquarello, L. Paulatto, C. Sbraccia, S. Scandolo, G. Sclauzero, A.P. Seitsonen, A. Smogunov, P. Umari, R.M. Wentzcovitch, QUANTUM ESPRESSO: a modular and open-source software project for quantum simulations of materials, J. Phys.: Condens. Matter, 21 (2009) 395502.

[2] P. Giannozzi, O. Andreussi, T. Brumme, O. Bunau, M. Buongiorno Nardelli, M. Calandra, R. Car, C. Cavazzoni, D. Ceresoli, M. Cococcioni, N. Colonna, I. Carnimeo, A. Dal Corso, S. de Gironcoli, P. Delugas, R.A. DiStasio, Jr., A. Ferretti, A. Floris, G. Fratesi, G. Fugallo, R. Gebauer, U. Gerstmann, F. Giustino, T. Gorni, J. Jia, M. Kawamura, H.Y. Ko, A. Kokalj, E. Kucukbenli, M. Lazzeri, M. Marsili, N. Marzari, F. Mauri, N.L. Nguyen, H.V. Nguyen, A. Otero-de-la-Roza, L. Paulatto, S. Ponce, D. Rocca, R. Sabatini, B. Santra, M. Schlipf, A.P. Seitsonen, A. Smogunov, I. Timrov, T. Thonhauser, P. Umari, N. Vast, X. Wu, S. Baroni, Advanced capabilities for materials modelling with Quantum ESPRESSO, J Phys Condens Matter, 29 (2017) 465901.

[3] J.P. Perdew, K. Burke, M. Ernzerhof, Generalized Gradient Approximation Made Simple, Physical Review Letters, 77 (1996) 3865-3868.


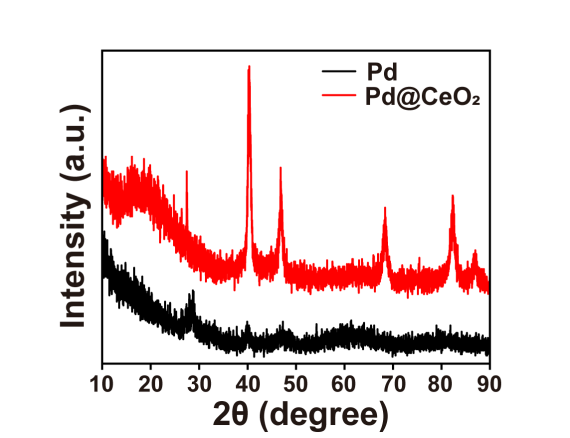


Figure S1. XRD of Pd nanocubes and Pd@CeO_2_ nanozymes.


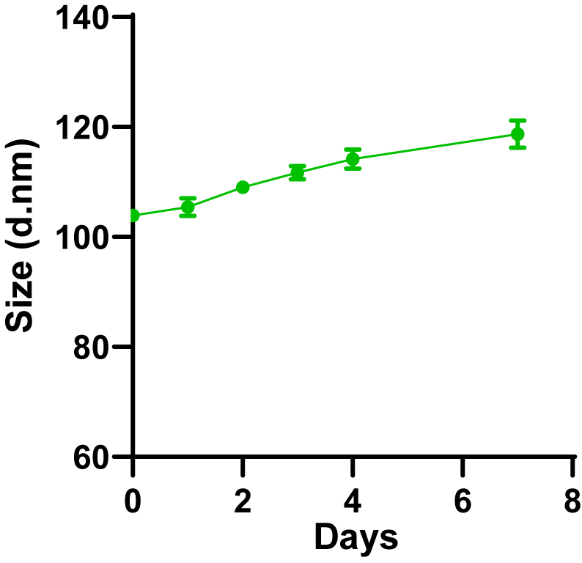


Figure S2. Size changes of Pd@CeO_2_ in PBS over 7 days, measured by DLS (n = 3).


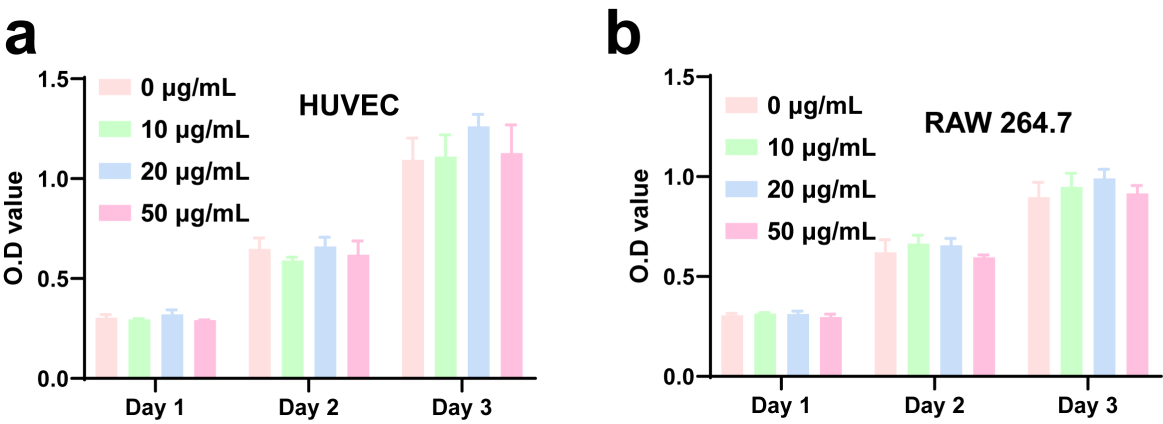


Figure S3. Effects of varying Pd@CeO_2_ concentrations on the proliferation of (a) HUVECs and (b) RAW 264.7. All data are presented as mean values with n = 3.


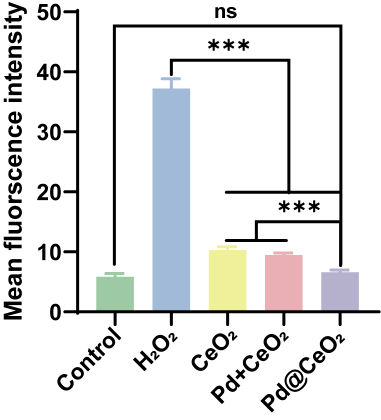


Figure S4. Flow cytometric analysis of mean fluorescence intensity in HUVECs under different treatment conditions (n = 3).


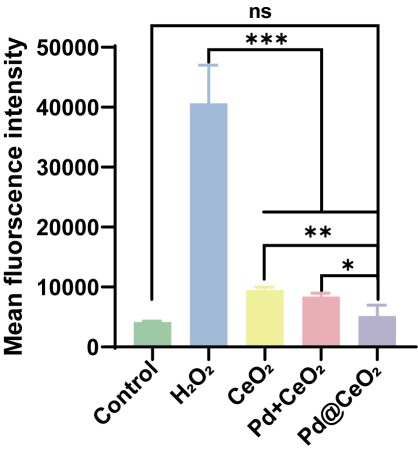


Figure S5. Semi-quantitative evaluation corresponding to manuscript Figure 3i (n = 3).


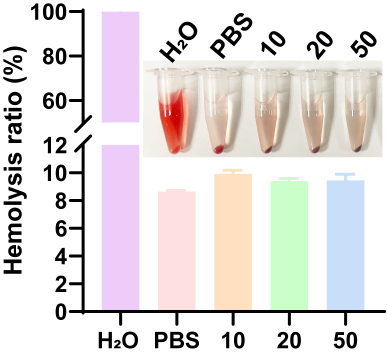


Figure S6. Hemolysis assay of red blood cells exposed to different treatments. H_2_O (positive control), PBS (negative control), and Pd@CeO_2_ of 10, 20, and 50 μg/mL (n = 3).


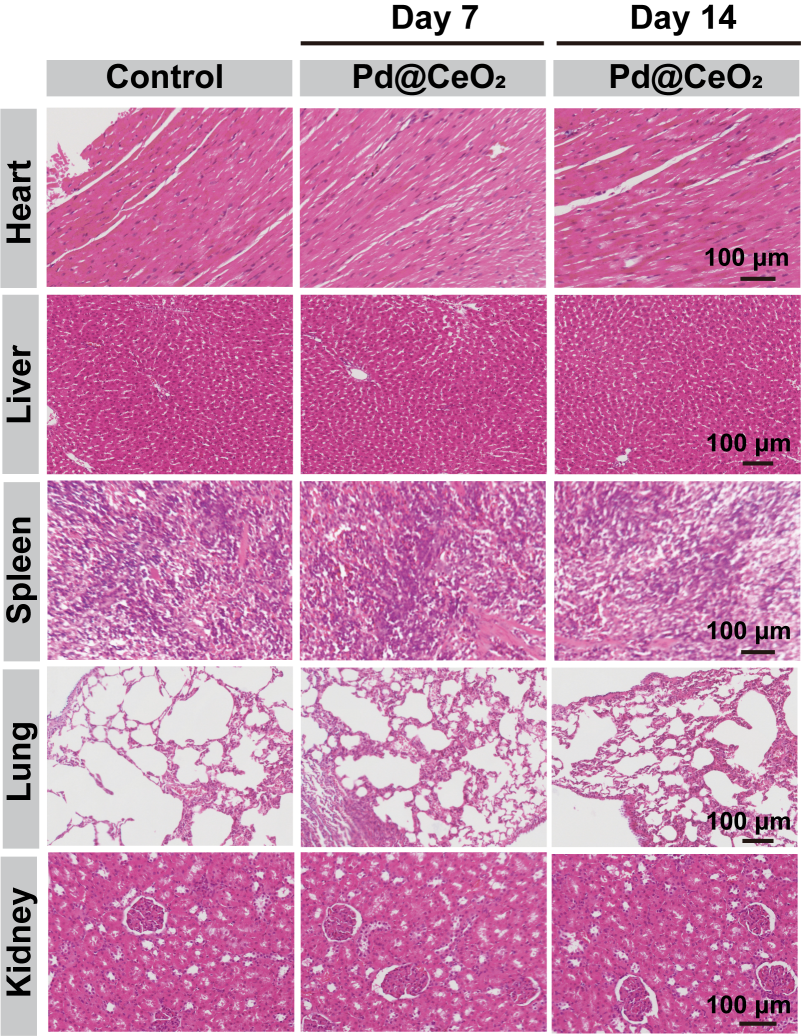


Figure S7. Representative H&E-stained sections of the heart, liver, spleen, lung, and kidney collected at Day 7 and Day 14 from control and Pd@CeO_2_-treated groups.

**Table S1 Hematologic analysis of different groups (Mean ± SEM, n = 3)**

| **Group** | **WBC (10****^9^/L)** | **RBC (10^12^/L)** | **HGB (g/L)** | **PLT (10****^9^/L)** |
| --- | --- | --- | --- | --- |
| I/R | 5.10 ± 0.59 | 9.12 ± 0.11 | 121.22 ± 2.78 | 500.21 ± 21.45 |
| Saline | 4.99 ± 1.21 | 9.26 ± 1.12 | 129.56 ± 4.36 | 567.27 ± 28.77 |
| CeO_2_ | 5.27 ± 1.80 | 8.93 ± 1.24 | 132.78 ± 2.80 | 489.56 ± 67.33 |
| Pd+CeO_2_ | 4.89 ± 0.32 | 8.78 ± 0.56 | 140.45 ± 4.89 | 467.78 ± 41.36 |
| Pd@CeO_2_ | 4.90 ± 2.16 | 9.14 ± 0.24 | 137.78 ± 3.11 | 521.27 ± 12.45 |

White blood cell (WBC), red blood cell count (RBC), haemoglobin (HGB), platelets (PLT).

**Table S2 Blood chemistry analysis of of different groups (Mean ± SEM, n = 3)**

| **Group** | **ALT (U/L)** | **AST (U/L)** | **BUN (mg/dL)** | **CREA (μmol/L)** |
| --- | --- | --- | --- | --- |
| I/R | 68.25 ± 1.21 | 189.24 ± 21.90 | 13.32 ± 7.56 | 30.23 ± 17.32 |
| CeO_2_ | 72.78 ± 3.45 | 190.25 ± 45.03 | 14.67 ± 3.66 | 34.76 ± 2.84 |
| Saline | 71.26 ± 8.77 | 154.75 ± 37.74 | 13.33 ± 1.91 | 39.86 ± 9.56 |
| Pd+CeO_2_  Pd@CeO_2_ | 77.34 ± 3.21  69.67 ± 11.02 | 178.83 ± 12.73  156.49 ± 44.23 | 14.37 ± 6.88  12.33 ± 12.12 | 29.55 ± 3.17  37.63 ± 11.77 |

Alanine aminotransferase (ALT), aspartate aminotransferase (AST), urea (UREA), Creatinine (CREA)**.**
